# The Extent and Phenotype of Coronary Artery Disease in Diabetes and Prediabetes: Insights From Imaging Studies and Potential Therapeutic Implications

**DOI:** 10.31083/RCM47550

**Published:** 2026-02-13

**Authors:** Filippo Luca Gurgoglione, Rebecca Navacchi, Alessia Ristagno, Giorgio Benatti, Emilia Solinas, Iacopo Tadonio, Andrea Denegri, Davide Donelli, Giulia Magnani, Laura Torlai Triglia, Michele Bianconcini, Federico Barocelli, Marco Covani, Mattia De Gregorio, Alessandra Dei Cas, Riccardo C. Bonadonna, Luigi Vignali, Giampaolo Niccoli

**Affiliations:** ^1^Division of Cardiology, University of Parma, Parma University Hospital, 43126 Parma, Italy; ^2^Endocrinology and Metabolic Diseases, Azienda Ospedaliero-Universitaria of Parma, 43126 Parma, Italy; ^3^Department of Medicine and Surgery, University of Parma, 43126 Parma, Italy; ^4^Endocrinology, Diabetology and Metabolic Diseases, University of Verona and University Hospital of Verona, 37134 Verona, Italy

**Keywords:** diabetes, prediabetes, coronary artery disease, plaque vulnerability, intracoronary imaging, SGLT2i, GLP1-RA

## Abstract

Type 2 diabetes mellitus (T2DM) is a complex metabolic disorder that is associated with a markedly increased risk of coronary artery disease (CAD) and cardiovascular (CV) mortality compared with the general population. Prediabetes, a heterogeneous intermediate glycemic state defined by impaired fasting glucose (IFG) and/or impaired glucose tolerance (IGT), and/or glycated hemoglobin (HbA1c) levels between 5.7% and 6.4%, is likewise associated with a significantly higher CV risk than normoglycemia. Over the past decade, both overall CAD burden and specific plaque morphologic features have been established as robust predictors of future adverse CV events using invasive and non-invasive coronary imaging modalities. More recently, growing evidence has highlighted the influence of glycemic abnormalities on the extent, progression, and phenotype of CAD, underscoring the interplay between metabolic dysfunction and atherosclerotic vulnerability. Therefore, this review aims to (i) elucidate the pathophysiological mechanisms linking T2DM and prediabetes with atherogenesis, (ii) summarize findings from coronary imaging studies in these populations, and (iii) evaluate therapeutic strategies designed to promote plaque stabilization and regression.

## 1. Introduction

Coronary artery disease (CAD) is the leading cause of mortality worldwide [1], arising from a multifaceted interplay of traditional and non-traditional cardiovascular (CV) risk factors [2].

Type 2 diabetes mellitus (T2DM) plays a pivotal role in both the initiation and progression of atherosclerosis and is associated with a two- to four-fold higher risk of CAD [3, 4] and nearly a 40% increase in CV mortality [5, 6, 7].

Prediabetes, a heterogeneous intermediate glycemic state characterized by impaired fasting glucose (IFG) and/or impaired glucose tolerance (IGT), and/or glycated hemoglobin (HbA1c) levels between 5.7% and 6.4%, also confers a significantly greater CV risk compared with normoglycemia [8, 9, 10].

Over the past years, advances in intracoronary imaging, including intravascular ultrasound (IVUS) [11], optical coherence tomography (OCT) [12], near-infrared spectroscopy (NIRS) [13], and coronary computed tomography angiography (CCTA) [14], have enhanced our understanding of CAD pathophysiology [15, 16, 17]. These techniques have consistently demonstrated that so-called vulnerable plaques, characterized by a thin fibrous cap (FC), large lipid core, and macrophage infiltration, together with overall CAD burden, are powerful predictors of future major adverse cardiovascular events (MACE) [18]. Importantly, longitudinal imaging studies have shown that targeted pharmacological interventions can stabilize high-risk plaques and, even promote regression of coronary atherosclerosis [19].

Recently, a growing body of evidence has investigated the impact of glycemic abnormalities on CAD extent and phenotype.

A deeper understanding of the molecular pathways underlying atherosclerosis, and how these relate to CAD extent and plaque phenotype, together with the effects of anti-inflammatory, lipid-lowering and anti-diabetic agents, may serve a threefold purpose: (i) improving our knowledge of the pathophysiology of coronary atherosclerosis; (ii) evaluating the direct and indirect impact of emerging pharmacological therapies; (iii) identifying new molecular pathways that could represent potential future therapeutic targets. This review aims to discuss the pathophysiological mechanisms linking T2DM and prediabetes to atherogenesis, summarize coronary imaging findings in these populations, and evaluate therapeutic strategies aimed at plaque stabilization and regression.

## 2. Pathophysiology of CAD in T2DM and Prediabetes

The excess CV risk conferred by T2DM and prediabetes stems from the interplay of conventional CV risk factors (hypertension, obesity, dyslipidemia, smoking habit) [2], and diabetes-specific metabolic disturbances [3]. Hyperglycemia and insulin resistance (IR) promote the formation of advanced glycation end products, oxidative stress, and activation of protein kinase C pathways [20]. These processes impair endothelial function, fostering a proinflammatory and vasoconstrictive state through reduced nitric oxide bioavailability and enhanced endothelin-1 expression [21]. In the early stages of atherogenesis, hyperglycemia and IR stimulate vascular smooth muscle cells proliferation, whereas in more advanced lesions, glycated and oxidized lipoproteins induce their apoptosis, contributing to FC thinning and plaque vulnerability [15]. IR further drives diabetic dyslipidemia, characterized by elevated very-low-density lipoprotein and low-density lipoprotein, and reduced high-density lipoprotein cholesterol, further amplifying vascular inflammation [22, 23]. Beyond these metabolic alterations, chronic inflammation plays a central role. Hyperglycemia and IR activate the NLRP3 inflammasome, promoting the release of interleukin-1β and -18, thereby enhancing macrophage recruitment, contributing to foam cell formation, and cytokine release [24]. Finally, glycemic abnormalities induce a prothrombotic milieu, characterized by enhanced platelet activation, increased procoagulant factors, and impaired fibrinolysis [25].

## 3. Coronary Atherosclerosis Imaging Modalities

### 3.1 Non-invasive Imaging Modalities

**Fig. 1.**
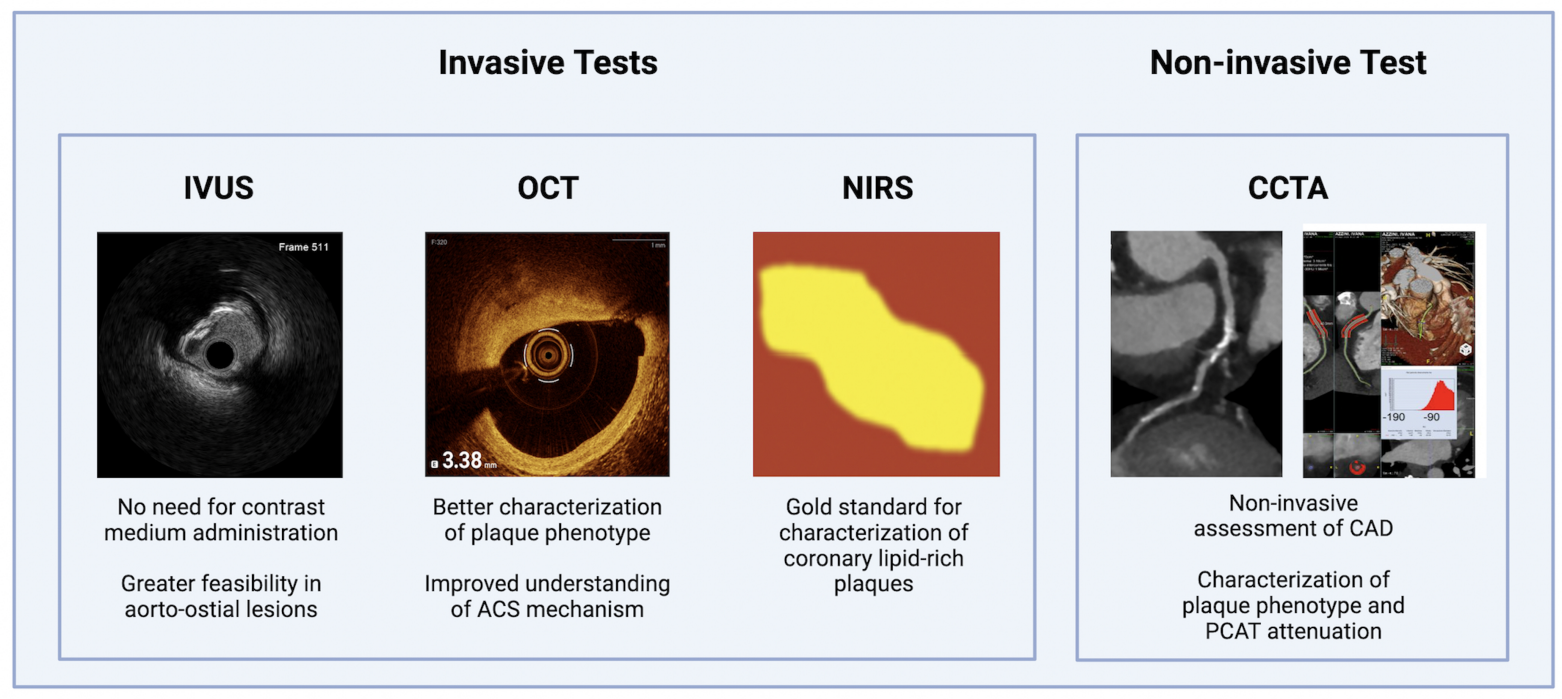
**Main relevant non-invasive and invasive coronary imaging tools**. CAD, coronary artery disease; CCTA, coronary computed tomography angiography; IVUS, intravascular ultrasound; NIRS, near-infrared spectroscopy; OCT, optical coherence tomography; PCAT, pericoronary adipose tissue attenuation.

Several non-invasive and invasive imaging modalities have been developed over time to assess the extent of CAD and the phenotype of atherosclerotic lesions. Understanding the fundamental principles of these techniques is essential to appreciate the advantages and limitations of each modality and to define their role in clinical practice (Fig. 1).

CCTA is a non-invasive imaging modality that enables evaluation of luminal stenosis and plaque phenotype. CCTA-derived high-risk plaque features, including low-attenuation plaque (LAP), positive remodeling, spotty calcifications, and the napkin-ring sign, strongly predict acute coronary events [14, 26, 27, 28]. CCTA also enables evaluation of pericoronary adipose tissue (PCAT), a marker of local vascular inflammation, that has been associated with adverse outcomes [29, 30, 31]. Current European Society of Cardiology and American Heart Association/American College of Cardiology guidelines endorse CCTA as a first-line diagnostic tool in patients with a low or moderate clinical likelihood of obstructive CAD with suspected chronic coronary syndrome (CCS) [32, 33].

### 3.2 Invasive Imaging Modalities 

#### 3.2.1 Intravascular Ultrasound (IVUS)

IVUS uses high-frequency ultrasound waves to generate cross-sectional images of the vessel wall, allowing precise quantification of lumen dimensions, plaque burden and phenotype. Virtual Histology IVUS applies spectral analysis of radiofrequency backscatter to classify plaque components into fibrous, fibro-fatty, necrotic core (NC), and dense calcium [11]. IVUS is able to identify thin-cap fibroatheromas (TCFAs), defined by a large NC and thin FC [11]. The landmark PROSPECT trial demonstrated that both extensive plaque burden and TCFA can predict future adverse CV events [11, 18].

#### 3.2.2 Optical Coherence Tomography (OCT)

OCT employs near-infrared light to generate high-resolution cross-sectional images (10–20 µm) of the coronary artery, offering an “*in vivo* biopsy” of atherosclerotic lesions. It is considered the gold standard for evaluating plaque vulnerability, with the ability to assess FC thickness, lipid arc, macrophage infiltration, neovascularization, and cholesterol crystals [12]. The CLIMA study demonstrated that the combination of FC thickness <65 µm, large lipid burden, and macrophage infiltration independently predicts MACE [34].

#### 3.2.3 Near-Infrared Spectroscopy (NIRS)

NIRS evaluates plaque composition by detecting lipid content through spectral absorption. The maximum lipid core burden index over 4 mm (maxLCBI_4⁢mm_) quantifies lipid extent and density, with higher values strongly associated with plaque vulnerability and adverse outcomes [13, 35, 36, 37].

## 4. T2DM: CAD Extent, Phenotype and Progression

The first evidence linking dysglycemia to CAD characteristics emerged from studies conducted in patients with diabetes, using both non-invasive and invasive imaging modalities.

### 4.1 Evidence From CCTA Studies 

Multiple CCTA studies have consistently shown that individuals with T2DM exhibit a greater burden and complexity of coronary atherosclerosis compared with non-diabetic controls [38].

In a prospective matched cohort study of 181 asymptomatic young adults (aged 25–40 years), subclinical CAD was significantly more prevalent in T2DM (58% vs. 20%, *p* < 0.001), with a nearly threefold higher relative risk after adjustment for CV risk factors compared to non—diabetic patients [39]. Similar findings have been confirmed in older cohorts, where diabetes was associated with a 1.4–2.1-fold higher risk of obstructive CAD, and a nearly doubled rate of multivessel disease, as well as left main or three-vessel involvement [40, 41, 42].

The SCOT-HEART trial further supported these observations, demonstrating higher proportions of both calcified plaque and LAP in patients with diabetes compared to controls, with a significantly increased risk of myocardial infarction (hazard ratio [HR]: 1.85) [43]. Notably, mortality among diabetic patients with non-obstructive CAD was comparable to that of non-diabetic patients with obstructive single-vessel disease [40]. This excess risk has been attributed to the high prevalence of vulnerable plaques in T2DM. Indeed, LAP content emerged as the strongest predictor of acute coronary syndrome (ACS) over 9.2 years of follow-up [44].

Longitudinal CCTA studies have consistently shown accelerated CAD progression in diabetes. In a U.S. propensity-matched cohort of 142 patients, T2DM patients experienced more than double the increase in total plaque volume compared with non-diabetics, driven largely by fibrofatty and low-attenuation components at 3.4 years [45]. The PARADIGM study extended these findings, showing greater progression of total plaque volume and a higher prevalence of vulnerable features, such as NC (21.5 ​± ​90.5% vs. –7.0 ​± ​35.8%, *p* ​= ​0.001), LAP (11.9% vs. 8.7%; *p* = 0.029), and spotty calcifications (12.3% vs. 9.1%; *p* = 0.020) at 3.8 years [46, 47]. Importantly, LAP exhibited nearly double the annualized progression rate compared with fibrous plaques [47].

Glycemic control and glycemic variability, as measured by the mean amplitude of glycemic excursions (MAGE), correlated with vulnerable plaque features on CCTA, with patients exhibiting multivessel vulnerability showing the highest variability [48].

Collectively, these findings underscore that T2DM is associated not only with greater plaque burden but also with accelerated progression and a shift toward high-risk phenotypes, offering a mechanistic explanation for the markedly elevated CV risk in this population.

### 4.2 Evidence From Intracoronary Imaging Studies

Invasive imaging studies have provided complementary insights, showing that patients with T2DM typically present with a greater coronary atherosclerotic burden and a higher prevalence of vulnerable plaque features compared with non-diabetic individuals. Collectively, these findings support the concept of a “pancoronary” vulnerability in diabetes, with high-risk characteristics in both culprit and NCLs (Table 1, Ref. [39, 42, 43, 45, 46, 49, 50, 51, 52, 53, 54]).

**Table 1.** **Summary of most relevant imaging studies evaluating CAD characteristics in patients with T2DM**.

First author, date, reference	Study design	Study population	Imaging tool	Imaging results	Clinical results
Nezarat, 2017 [39]	Observational, matched study	181 asymptomatic young (25–40 years old) patients	CCTA	T2DM patients had a significantly higher prevalence of subclinical CAD (58% vs. 20%, *p * < 0.001). and a higher total plaque burden (risk ratio 2.887, *p * < 0.001) compared to non-T2DM patients.	NA
Rana, 2012 [42]	Observational, matched study	10.110 adult patients (≥18 years old) with suspected CAD	CCTA	T2DM patients had higher rates of obstructive CAD (37% vs. 27%, *p * < 0.0001) and obstructive three-vessel disease (9 vs. 5%, *p * < 0.0001).	Risk of mortality was higher for T2DM patients across the full spectrum of CAD.
Gebert, 2025 [43]	Post-hoc analysis of a RCT	1.769 adult patients (18–75 year old) with CCS	CCTA	DM patients had higher calcified, non-calcified, low attenuation and total plaque burdens. DM was an independent predictor of calcified plaque burden (*p* = 0.009).	DM was associated with an increased risk of myocardial infarction (HR 1.85, *p* = 0.024).
Nakanishi, 2016 [45]	Observational, matched study	142 adults patients clinically referred for serial CCTA	CCTA	DM patients showed a 2-fold greater progression in normalized TPV than non-diabetes patients (118.3 mm^3^, vs. 52.8 mm^3^, *p* = 0.005), especially LAP and fibrous-fatty plaques, at 3.4 years.	NA
Kim, 2018 [46]	Observational matched study	1602 adults patients who underwent 2 or more clinically indicated CCTA within two years	CCTA	DM patients had greater progression of TPV and a higher prevalence of vulnerable features, such as LAP (11.9% vs. 8.7%; *p* = 0.029) and spotty calcifications (12.3% vs. 9.1%; *p* = 0.020) at 3.8 years.	NA
Nasu, 2008 [49]	Observational	90 adults patients with CCS	IVUS	DM patients showed an increased amount of intraplaque dense calcium and necrotic core (NC) and nearly double the prevalence of TCFA compared with non-diabetics (75% vs. 41%; *p* = 0.001).	NA
Araki, 2012 [50]	Observational	146 adults patients with CCS	IVUS	DM patients had large lipidic (8.39 ± 3.38% vs. 5.25 ± 2.30%, *p * < 0.0001) and necrotic (23.65 ± 11.54% vs. 12.99 ± 10.71%, *p * < 0.0001) plaques at culprit lesion sites.	NA
Kato, 2012 [51]	Observational	108 adults patients with CCS who underwent 3-vessel OCT	OCT	DM patients had a larger lipid index and and higher prevalence of calciﬁcation (72.2% vs. 48.4%, *p* = 0.034). Patients with DM and HbA1c ≥8% also had the highest prevalence of TCFA and macrophage inﬁltration.	NA
Sugiyama, 2018 [52]	Observational	322 adults patients with ACS who undewent preintervention OCT imaging of the culprit plaque	OCT	DM patients had a higher prevalence of lipid-rich plaque (58.9% vs. 44.9%, *p* = 0.030) and macrophage accumulation (60.0% vs. 44.9%, *p* = 0.019) in the culprit plaques; they had also greater lipid arc, thinner FCT, and more frequent TCFA in the non-culprit plaques.	NA
Kedhi, 2021 [53]	Observational	390 T2DM patients with at least one non-ischemic non-culprit lesion	OCT	DM patients showed a higher proportion of vulnerable non-culprit lesions: TCFA represented 25% of FFR-negative lesions.	OCT-detected TCFA was associated with a 5-fold higher rate of MACE at 18 months (HR: 5.12).
Gyldenkerne, 2023 [54]	Observational	898 adults patients with ACS	NIRS-IVUS	The prevalence of high-risk plaques was similar independent of DM in bith culprit (maximum plaque burden ≥70%: 90% vs. 93%, *p* = 0.34; maximum lipid core burden index ≥324.7: 66% vs. 70%, *p* = 0.49) and nonculprit lesions (maximum plaque burden ≥70%: 23% vs. 22%, *p* = 0.37; maximum lipid core burden index ≥324.7: 26% vs. 24%, *p* = 0.47).	DM was associated with an ≈2-fold increased rate of MACE during a median 3.7-year follow-up.

ACS, acute coronary syndrome; CCS, chronic coronary syndrome; DM, diabetes mellitus; FCT, Fibrous cap thickness; HbA1c, glycated hemoglobin; LAP, low-attenuation plaque; MACE, major adverse cardiovascular events; NA, not available; RCT, randomized clinical trial; TCFA, thin cap fibroatheroma; TPV, total plaque volume; NC, necrotic core; HR, hazard ratio.

In patients with CCS, diabetes has been associated with larger lipidic and NC volumes, greater calcium content and more frequent negative vessel remodeling [48, 49, 50, 55]. IVUS studies confirmed that plaques in diabetics are more often vulnerable, with nearly double the prevalence of TCFA compared with non-diabetics (75% vs. 41%; *p* = 0.001) [50]. Moreover, insulin-dependent patients displayed larger NCs than those with non–insulin-dependent diabetes, underscoring the link between poor glycemic control and plaque vulnerability [56].

Similar findings emerged in ACS. In a cohort of 147 ACS patients, those with T2DM had higher rates of multivessel disease, greater plaque burden and more frequent TCFAs (60% vs. 42%, *p* = 0.003) compared with non-diabetics. They also exhibited higher hs-C reactive protein levels, reinforcing the contribution of systemic inflammation to diabetic CAD [57]. The landmark PROSPECT trial confirmed these associations, showing that diabetics had plaques with greater NC and calcium content, and experienced higher 3-year rates of MACE compared with controls (29.4% vs. 17.4%, *p* = 0.03) [58].

OCT studies provided complementary mechanistic insights. In CCS patients, three-vessel OCT demonstrated significantly greater plaque vulnerability in T2DM, with poor glycemic control (HbA1c ≥8.0%) linked to higher TCFA prevalence (17.2% vs. 6.3%, *p* = 0.031) [51]. In ACS, T2DM patients displayed more vulnerable features in both culprit and NCLs, including greater maximal lipid arc, thinner FC, and higher TCFA prevalence [52, 59].

Beyond chronic hyperglycemia, glycemic variability also emerged as a key driver: in a IVUS study of 57 ACS patients, higher MAGE correlated with larger plaque volume and greater lipid content [60], while an OCT study identified MAGE as the strongest predictor of lipid index, thin FC, and TCFA presence, highlighting the destabilizing effect of glycemic fluctuations [61].

A post-hoc analysis of the COMBINE OCT–FFR study including 390 T2DM patients with at least one non-ischemic NCL showed that OCT-defined TCFA was the strongest predictor of MACE at 18 months (HR: 5.12) [53]. In contrast, the PROSPECT II trial did not observe significant differences in plaque features between ACS patients with and without diabetes using NIRS–IVUS, in line with two smaller OCT studies [54, 62, 63]. The high prevalence of statin treatment, with optimal lipids and glycemic control may have attenuated the differences. However, MACE occurred more frequently in patients with T2DM diabetes at 3.7 years, primarily attributable to increased risk of myocardial infarction [63]. Several longitudinal studies consistently highlight the adverse impact of diabetes on CAD progression and vulnerability, which likely explains the worse prognosis observed in T2DM.

A pooled analysis of over 2200 CCS patients revealed significantly higher percent and total atheroma volumes in T2DM [64]. Similarly, a PRECISE trial subanalysis identified diabetes as an independent predictor of plaque progression and of new TCFAs at follow-up (20.3% vs. 12.5%, *p* = 0.01). NCL–related MACE were also more frequent in diabetics at 3 years (9.5% vs. 1.7%, *p* = 0.027) [65]. Importantly, HbA1c levels correlated with greater annualized plaque progression and worse clinical outcomes, underscoring the pivotal role of glycemic control [66, 67]. Landmark trials have demonstrated that CV risk in T2DM is determined not only by hyperglycemia, but also by a complex phenotype including disease duration, degree of IR, microvascular complications, and established CVD. Notably, the insulin-resistantand the microvascular complication–related phenotypes exhibit the highest CV vulnerability [68].

It is important to emphasize, however, that T2DM is a heterogeneous condition. Clinical, genetic, and laboratory variables define distinct T2DM phenotypes, with differences in etiology, natural history, and prognosis [69]. In this regard, Montone *et al*. [70] studied 320 T2DM patients stratified by the presence of diabetes-related microvascular complications (DMC: any of retinopathy, neuropathy, or nephropathy). Patients with DMC exhibited a higher prevalence of multivessel disease and, at OCT analysis, a greater burden of large calcifications but a lower prevalence of lipid-rich lesions, suggesting more severe but morphologically more stable CAD. From a clinical standpoint, DMC patients showed worse glycemic control but a lower prevalence of metabolic syndrome traits, supporting the existence of heterogeneous pathways of CAD development and progression in diabetes [70, 71]. Such phenotypic differences, together with variations in diabetes duration, ay, at least in part, explain the heterogeneity observed between imaging studies.

## 5. Prediabetes: CAD Extent, Phenotype and Progression

In line with the growing scientific interest in prediabetes, driven by evidence of its adverse prognosis compared with normoglycemia, several non-invasive and invasive imaging studies have been conducted to elucidate the mechanisms underlying this increased risk and to identify the potential role of antidiabetic therapies in preventing or reversing prediabetes.

### 5.1 Evidence From CCTA Studies

CCTA studies consistently demonstrate that individuals with prediabetes exhibit a greater extent of CAD and a higher prevalence of high-risk plaque features compared with normoglycemic patients, with patterns resembling those observed in DM. Definitions of prediabetes, however, varies across studies, including IFG, IGT, elevated HbA1c, or IR assessed by Homeostasis Model Assessment (HOMA) index or the triglyceride-glucose (TyG) index. In a U.S. observational study of 216 asymptomatic participants without prior CAD, patients were stratified into DM (n = 52), IFG (n = 44), and normoglycemia (n = 120). The prevalence of obstructive CAD (>60% luminal stenosis) was significantly higher in DM (36.5%) and IFG (29.5%) compared with normoglycemia (13.3%; *p* = 0.001), as was the frequency of non-calcified lesions (DM 6.3%, IFG 6.7%) [72].

A Danish prospective study of 148 patients further highlighted the impact of prediabetes on plaque vulnerability. Those with IGT (n = 80) had a greater volume of LAP compared with normal glucose tolerance, a higher prevalence of the napkin-ring sign (12% vs. 5%; *p* = 0.02), and a trend towards more frequent spotty calcifications (32% vs. 23%, *p* = 0.10) [73].

The Miami Heart Study (MiHeart), including 2352 participants without atherosclerotic CV disease stratified by HbA1c, confirmed these findings. Both prediabetes and T2DM were independently associated with higher odds of any CAD lesions (1.30 and 1.75, respectively), as well as plaques with ≥1 high-risk feature (OR 1.65 and 2.53, respectively) [74].

Beyond glycemia, IR emerged as a key determinant of CAD burden and progression. In a sub-study of the DANCAVAS trial of asymptomatic men aged 65–75 years without known diabetes, those in the highest HOMA-IR tertile had greater necrotic plaque (*p* = 0.02) and fibrous-fatty plaque volumes (*p* = 0.01), with reduced fibrotic plaque burden (*p* < 0.001) [75].

The PARADIGM study further demonstrated the prognostic role of the TyG index, a surrogate of IR. Among 1143 subjects undergoing serial CCTA, plaque progression, defined as the any increase in plaque volume between baseline and follow-up, was significantly greater in the highest TyG tertile, with an adjusted odd ratio of 1.65 [76].

Additional mechanistic insights were provided by a Chinese single-center study of 569 participants, which found that the TyG index was independently associated with PCAT attenuation in both prediabetic and T2DM subgroups. Importantly, TyG index and PCAT attenuation acted synergistically in determining CAD severity, with mediation analysis showing that PCAT attenuation partially explained the association between TyG and multivessel CAD. These findings suggest that increased PCAT attenuation may represent a pathophysiological link between IR and higher CAD risk, and potentially a novel therapeutic target [77]. Among prediabetes phenotypes, IGT confers the highest CV risk, largely due to sustained postprandial hyperglycemia, which promotes oxidative stress, endothelial dysfunction, and vascular inflammation. This excess risk is thought to reflect underlying peripheral (skeletal muscle) IR and impaired first-phase insulin secretion, both of which exacerbate postprandial glycemic excursions. In contrast, TFG and HbA1c-defined dysglycemia are associated with intermediate risk profiles, although the prognostic value of HbA1c in the prediabetic range remains heterogeneous across studies [78]. Achieving regression to normal glucose regulation or maintaining IGT following intervention significantly reduces the risk of CV and microvascular complications, with benefits proportional to the duration of the non-diabetic state; this legacy effect, driven by lower cumulative exposure to chronic hyperglycemia, highlights the importance of preventive strategies aimed not only at halting progression to diabetes but also at sustaining NGR to maximize long-term vascular protection [79].

### 5.2 Evidence From Intracoronary Imaging Studies

Intracoronary imaging studies consistently associate prediabetes with more extensive CAD and high-risk plaque features across the spectrum of CAD presentations (Table 2, Ref. [72, 73, 74, 75, 76, 80, 81, 82, 83, 84, 85]).

**Table 2.** **Summary of most relevant imaging studies evaluating CAD characteristics in patients with prediabetes**.

First author, date, reference	Study design	Study population	Imaging tool	Imaging results	Clinical implications
Gurudevan, 2016 [72]	Observational	216 asymptomatic patients without prior CAD	CCTA	The prevalence of obstructive CAD (>60% luminal stenosis) was significantly higher in DM (36.5%) and IFG (29.5%) compared with normoglycemia (13.3%; *p* = 0.0015).	Both pre-diabetes and DM had a higher extent of CAD comapred with normoglycemia.
Andersen, 2025 [73]	Observational	148 adults patients with suspected CCS	CCTA	Patients with IGT had a significantly higher prevalence of LAP (60% vs .42%, *p* = 0.007) and napkin-ring sign (12% vs. 5%, *p* = 0.02) compared with NGT.	The prevalence of high-risk plaques is significantly higher in pre-diabetes compared with normoglycemia.
Patel, 2023 [74]	Observational	2352 asymptomatic patients without prior CAD	CCTA	Both prediabetes and T2DM were independently associated with higher odds of any CAD lesions [1.30 (*p* = 0.02) and 1.75 (*p* = 0.005), respectively], as well as plaques with ≥1 high-risk feature (OR 1.65 and 2.53, respectively) compared with NGT.	Pre-diabetes is associated with a higher extent of CAD, with vulnerable features, compared with normoglycemia.
Larsson, 2023 [75]	Sub-study of a RCT	450 asymptomatic men without known diabetes	CCTA	Patients in the higher H-IR tertile had higher median necrotic plaque volume (18.2 vs. 11.0 mm^3^, *p* = 0.02) and fibrous-fatty plaque volume (*p* = 0.01) and lower fibrotic plaque burden (*p * < 0.001) compared to lower H-IR tertile.	High-risk plaques are more common in pre-diabetes than normoglycemia.
Won, 2020 [76]	Observational	1143 adults patients who underwent serial CCTA	CCTA	Higher TyG index was independently associated with greater progression of coronary plaque volume (OR 1.65, *p* = 0.005). In addition, the TyG index had a positive association with the annual change of total PV, TAVnorm, and PAVtotal.	Pre-diabetes is associated with a higher risk of CAD progression at a median follow-up of 3.2 years.
Amano, 2008 [80]	Observational	165 adults patients with ACS or stable angina who underwent PCI	IVUS	IGR was positively associated with an increase in %LV (*p* = 0.02) and a decrease in %FV (*p* = 0.03) of coronary plaques. Patients in the highest tertile of H-IR had signiﬁcantly increased of LAP prevalence (*p* = 0.008).	The prevalence of high-risk plaques in ACS is similar between DM and pre-diabetes and significantly higher compared with normoglycemia.
Iguchi, 2014 [81]	Observational	155 patients undergoing OCT at culprit lesions (65% CCS, 35% ACS)	OCT	Patients in the higher H-IR tertile had more frequent prevalence of lipid-rich plaques than those in the middle and lower tertiles (83 vs. 62 vs. 57%; *p* = 0.01). In addition, a H-IR >2.5 was independently associated with TCFA (OR 2.68; *p* = 0.005).	The presence of TCFA is associated with H-IR and ACS presentation.
Mitsuhashi, 2011 [82]	Observational	82 non-diabetic adults patients with ACS	IVUS	Patients in the highest tertile of IR had larger lipid area (37.6 ± 16.6%) compared with intermediate (25.8 ± 11.9%) and lowest tertiles (27.5 ± 14.7%; *p * < 0.01).	Hyperinsulinemia is associated with a higher prevalence of high-risk plaques compared to normoglycemia.
Zhang, 2018 [83]	Observational	216 adults patients with ACS	OCT	Plaques in patients with raised HbA1c (5.7%–6.4%) had high risk features similar to DM such as longer lipid length (*p* = 0.004), greater lipid index (*p* = 0.001) and higher prevalence of calcification (38.7% vs. 26.3%, *p* = 0.048), whereas macrophage infiltration was more frequent in DM than prediabetes (20.5% vs. 11.8%; *p* = 0.067).	The prevalence of high-risk plaques in ACS is similar between DM and pre-diabetes and significantly higher compared with normoglycemia.
Wu, 2019 [84]	Observational	145 adults patients with ACS	OCT	Patients in the highest H-IR quartile had a threefold higher prevalence of TCFA ( *p* = 0.001) and macrophage infiltration (*p * < 0.001) and a sevenfold higher prevalence of spotty calcifications (*p * < 0.001) compared with the lowest quartile.	The presence of high-risk plaques is associated with H-IR.
Farhan, 2021 [85]	Observational	507 adults patients with ACS	IVUS	Patients with pre-diabetes and DM had a higher prevalence of echolucent plaques compared to patients with insulin sensitivity.	Pre-diabetes and DM were independently associated with increased risk of MACE compared with insulin sensitive (aHR 2.29, *p* = 0.01 for pre-diabetes and aHR 2.12, *p* = 0.009 for DM).

aHR, adjusted hazard ratio; DM, diabetes mellitus; FV, fibrous volume; H-IR, Homeostasis model assessment of insulin resistance; IFG, impaired fasting glucose; IGR, impaired glucose regulation; IGT, impaired glucose tolerance; LV, lipid volume; NGT, normal glucose tolerance; NSTEMI, non-ST elevation myocardial infarction; OR, odds ratio; PAVtotal, total percent atheroma volume; PCI, percutaneous coronary intervention; PV, plaque volume; TAVnorm, normalized total atheroma volume; TyG index, Ln [fasting triglyceride (TG) (mg/dL) × fasting blood glucose (FBG) (mg/dL)/2].

In patients with predominantly CCS, Amano *et al*. [80] evaluated 165 subjects (74.5% with CCS) undergoing IVUS assessment of the culprit lesion stratified into normoglycemia, impaired glucose regulation (including IFG and IGT, n = 44), and DM (n = 83), both DM and prediabetes exhibited significantly greater lipid volume percentage compared with normoglycemia.

Similarly, Iguchi *et al*. [81] analyzed 155 patients (65.5% with CCS) undergoing OCT at culprit lesions, stratified by tertiles of HOMA-IR. Those in the highest tertile had a higher prevalence of OCT-defined vulnerable features, including TCFA, microvessels, larger lipid arc, and thinner FC. A HOMA-IR >2.5 was independently associated with TCFA (OR 2.68; 95% CI, 1.34–5.41; *p* = 0.005) [81].

Two studies focused on ACS. Mitsuhashi *et al*. [82] examined 82 non-diabetic ACS patients stratified into tertiles of insulin response. Patients in the highest tertile had larger lipid area (37.6 ± 16.6%) compared with intermediate (25.8 ± 11.9%) and lowest tertiles (27.5 ± 14.7%; *p* < 0.01) [82]. Zhang *et al*. [83] assessed 305 NCLs from 216 ACS patients using OCT, stratifying by HbA1c. Compared with normoglycemia, prediabetes was associated with longer lipid length and greater lipid index, with no significant differences between prediabetes and DM. Macrophage infiltration was more frequent in DM than prediabetes (20.5% vs. 11.8%; *p* = 0.067), while other high-risk features were similar [83].

Further evidence links IR to plaque vulnerability. In a study of 145 ACS patients, those in the highest HOMA-IR quartile had a threefold higher prevalence of TCFA and macrophage infiltration and a sevenfold higher prevalence of spotty calcifications compared with the lowest quartile [84]. An elevated TyG index independently predicted TCFA in NCLs (OR 4.94 per 1-unit increase) [86]. Of interest, a post-hoc analysis of the PROSPECT trial found similar rates of high-risk plaque features when prediabetes was defined by fasting glucose or HbA1c. However, when defined by IR, non-diabetic patients with HOMA-IR ≥5 exhibited markedly higher rates of echolucent plaques, characterized by early lipid pool and NC formation, and a higher occurrence of MACE compared with HOMA-IR <2 (adjusted HR 2.29, *p* = 0.01) [85].

## 6. Therapeutic Implications

Over the past decade, landmark randomized trials have shown that targeting the two major drivers of atherogenesis—hyperlipidemia and inflammation—can induce regression and stabilization of CAD, ultimately translating into fewer adverse CV outcomes [87]. However, the presence of diabetes consistently attenuates the degree of plaque regression, with poor glycemic control emerging as a pivotal determinant of treatment efficacy [88, 89]. The SATURN trial demonstrated that patients with diabetes achieve a regression comparable to that of non-diabetic individuals only when cholesterol levels were significantly low and strict glycemic control was maintained [90].

Accumulating evidence now indicates that antidiabetic agents may exert direct anti-atherogenic effects, promoting plaque stabilization and even regression through mechanisms extending beyond glucose control [89] (Table 3, Ref. [91, 92, 93, 94, 95, 96]).

**Table 3.** **Mechanistic and imaging evidence supporting the anti-atherosclerotic actions of glucose-lowering therapies**.

Glucose-lowering agent	Study design	Imaging results	Pathophysilogical mechanisms
Metformin [91]	• Preclinical studies on ApoE/mice treated daily with metformin for 10 weeks	• Reduced aortic atherosclerotic plaque fomation.	• Reduced intraplaque recruitment of bone-marrow–derived monocytes through inhibition of Ccr2 expression.
	• Clinical study on diabetic and non-diabetic patients		• Inhibition of pro-inflammatory cytokines and reduction of the neutrophil-to-lymphocyte ratio, independent of diabetic status.
Pioglitazone [92]	• RCT in ACS patients without diabetes undergoing VH-IVUS • RCT in patients with prediabetes or diabetes and carotid atherosclerosis undergoing FDG-PET	• Plaques in pioglitazone-treated patients showed a significantly greater reduction in PAV, plaque burden, and TAV.	• Reduced systemic inflammation, as evidenced by lower levels of hs-CRP.
		• Pioglitazone reduced plaque inflammation compared with glimepiride.	
SGLT2i [93, 94, 95]	• Observational studies in patients with T2DM and CAD, presenting with either ACS or CCS, and undergoing CCTA or OCT assessment	• Plaques treated with SGLT2i therapy showed a significantly reduction in plaque volume, particularly within non-calcified plaques, reduced macrophage infiltration, smaller lipid arcs and calcific volume both in culprit and non-culprit lesions.	• Reduced intraplaque macrophage content by inhibiting macrophage polarization and pro-inflammatory cytokines.
			• Reduced lipid content through inhibition of foam-cell formation and leukocyte adhesion.
			• Reduced intraplaque calcification by inhibiting NLRP3 signaling pathway and endoplasmic-reticulum–stress–dependent thioredoxin domain activity.
			• Reduced prothrombotic state through inhibition of platelet activation.
GLP-1RA [96]	• RCT in diabetic patients with CAD undergoing PCI and NIRS-IVUS assessment	• GLP-1RA treated patients demonstrated a greater frequency of maxLCBI_4⁢mm_ regression.	• Enhanced polarization of macrophages toward anti-inflammatory M2 phenotype.

Ccr2, CC chemokine receptor 2; FDG-PET, 18F-fluorodeoxyglucose positron emission tomography; GLP-1RA, Glucagon-like peptide-1 receptor agonists; hs-CRP, high-sensitivity C-reactive protein; maxLCBI_4⁢mm_, maximum lipid-core burden index at 4-mm segment; NA, not available; NLRP3, nucleotide-binding domain leucine-rich repeat–containing pyrin domain protein-3; PAV, percent atheroma volume; PCI, percutaneous coronary intervention; RCT, randomized clinical trial; SGLT2i, Sodium–Glucose Cotransporter-2 inhibitors; TAV, total atheroma volume; VH-IVUS, intravascular ultrasonography with virtual histology.

Metformin may exert favorable effects on plaque phenotype. In a single-center U.S. study assessing 409 NCLs in 313 diabetic patients by OCT, metformin use at the time of imaging correlated with more stable characteristics, including smaller lipid arcs (median 163.4° vs. 193.5°, *p* = 0.02), and lower prevalence of cholesterol crystals (2.4% vs. 14.5%, *p* = 0.03) and spotty calcifications (6.5% vs. 23.2%, *p* = 0.01), even after adjustment for clinical, glycemic, and lipid variables [91].

Similar anti-atherogenic properties have been demonstrated with pioglitazone, a peroxisome proliferator-activated receptor-γ agonist. In the Dresden-PPP TRIAL, which enrolled 54 non-diabetic patients with ACS, 9 months of pioglitazone therapy resulted in a significant reduction in IVUS-derived NC content and a greater decrease in total plaque volume compared to placebo [92].

Sodium–Glucose Cotransporter-2 inhibitors (SGLT2i) reduce glucose reabsorption in the renal proximal tubules and confer CV protection through metabolic, hemodynamic, and anti-inflammatory mechanisms [97, 98]. In a CCTA study of 236 patients with T2DM and non-obstructive CAD, SGLT2i therapy was associated with a significant reduction in total plaque volume, particularly within non-calcified plaques, compared with matched controls [93]. Likewise, OCT analysis of 369 patients with CCS revealed that SGLT2i use was associated with features of enhanced plaque stability, including thicker FC, smaller lipid arcs, and reduced macrophage infiltration. These morphological changes translated into a lower incidence of MACE at follow-up (12 [10.8%] vs. 57 [22.1%]; *p* < 0.05), with consistent benefits across different SGLT2i, supporting a class effect [94]. Even in the setting of ACS, SGLT2i promoted stabilization of NCLs. After six months of therapy, OCT demonstrated significantly thicker FCs and greater reductions in lipid arc. These plaque-modifying effects were paralleled by lower rates of MACE and coronary revascularizations [95].

Glucagon-like peptide-1 receptor agonists (GLP-1RA) have also been shown to favorably modulate coronary atherosclerosis through both metabolic and direct vascular mechanismsIn a prespecified analysis of the OPTIMAL trial, NIRS/IVUS imaging revealed that GLP-1RA use was independently associated with regression of the maximum lipid core burden index over 48 weeks (85.6% vs. 42.0%; *p* = 0.01) [96].

## 7. Gaps in Knowledge and Future Perspectives

Given their complementary mechanisms of action, there is growing interest in the potential synergistic effects of combining SGLT2i and GLP-1RA in the management of CAD. In an observational matched study including 1.325 patients with ACS and T2DM who had been treated with SGLT2i for at least three months before admission, the addition of GLP-1RA was associated with a 31% lower risk of MACE at one year compared with SGLT2i alone (HR 0.69, 95% CI 0.49–0.98) [99]. Whether these agents provide additive protective effects against CAD, including when combined with proprotein convertase subtilisin/kexin type 9 (PCSK9) inhibitors, remains to be confirmed in dedicated randomized trials.

Tirzepatide, a dual GIP/GLP-1 RA, leverages synergistic metabolic effects by enhancing glucose-dependent insulin secretion and promoting negative energy balance [100, 101]. In a large real-world cohort of 140,308 patients with T2DM, tirzepatide use was associated with significantly lower risks of all-cause mortality and MACE compared with GLP-1RA [102]. The ongoing T-Plaque RCT will assess whether 52 weeks of tirzepatide therapy can halt or even reverse CAD progression. The study is enrolling 100 patients aged 40–80 years with HbA1c between 7.0% and 10.5% and at least two coronary plaques with >20% stenosis on CCTA, with the primary endpoint being the change in non-calcified plaque volume [103].

Beyond these pharmacological advances, several important questions remain unresolved. It is unclear whether the effects of anti-inflammatory [104], lipid-lowering, and glucose-lowering therapies on plaque stabilization differ across subgroups of patients with T2DM, such as those vs. without DMC.

Emerging evidence suggests that distinct pathophysiological pathways, ranging from predominant IR to impaired β-cell function, confer variable susceptibility to atherosclerotic burden and plaque vulnerability. Recognizing such heterogeneity is essential for advancing precision medicine, as it may allow the tailoring of therapeutic strategies to specific phenotypic profiles, ultimately improving CV outcomes in this high-risk population. Moreover, in prediabetes, the impact of available therapies on coronary plaque regression remains unknown. Distinct classifications and phenotypes of prediabetes, each with differing prevalence and prognostic implications, have been described [105, 106, 107]. However, it is plausible that early intervention in prediabetes may modify the trajectory of atherosclerosis by mitigating IR and vascular inflammation, thereby limiting plaque vulnerability and improving long-term CV outcomes. Future randomized trials are needed to clarify the relationship between prediabetes phenotypes and the stabilization or regression of coronary plaque burden and vulnerability.

## 8. Conclusions

Imaging studies demonstrate that both prediabetes and T2DM are associated with a more extensive atherosclerotic burden and a higher prevalence of vulnerable plaque features compared with normoglycemic individuals (Graphical Abstract). This is consistent in both asymptomatic patients with subclinical disease and those with established CAD.

Early detection of glycemic abnormalities and the timely implementation of comprehensive, individualized risk management are crucial to attenuate CAD progression and improve long-term outcomes. Such strategies should encompass stringent control of glycemia, lipid profile, and blood pressure, alongside targeted modulation of vascular inflammation.

## Abbreviations

ACS, acute coronary syndrome; CAD, coronary artery disease; CCS, chronic coronary syndrome; CCTA, coronary computed tomography angiography; CV, cardiovascular; DMC, diabetic microvascular complications; FC, fibrous cap; GLP-1RA, Glucagon-like peptide-1 receptor agonists; HbA1c, glycated hemoglobin; HOMA, Homeostasis Model Assessment; HR, hazard ratio; IFG, impaired fasting glucose; IGT, impaired glucose tolerance; IR, insulin resistance; IVUS, intravascular ultrasound; LAP, low-attenuation plaque; MACE, major adverse cardiovascular events; MAGE, mean amplitude of glycemic excursions; maxLCBI₄mm, maximum lipid core burden index over 4 mm; NC, necrotic core; NCL, non-culprit lesion; NIRS, near-infrared spectroscopy; OCT, optical coherence tomography; PCAT, pericoronary adipose tissue; SGLT2i, Sodium–Glucose Cotransporter-2 inhibitors; T2DM, type 2 diabetes mellitus; TCFA, thin-cap fibroatheroma; NC, necrotic core; TCFAs, thin-cap fibroatheromas; PCSK9, proprotein convertase subtilisin/kexin type 9.
